# Reproductive performance of dairy cows assigned to either a combination of estrus detection using sensors and timed artificial insemination or to a synchronization program

**DOI:** 10.3168/jdsc.2025-0779

**Published:** 2025-11-21

**Authors:** Alessio Valenza, Alex Bach

**Affiliations:** 1 Ceva Salute Animale, 20127 Milano, Italy; 2 Department of Animal Science, University of Lleida, 25198 Lleida, Spain; 3 Institució de Recerca i Estudis Avançats (ICREA), 08010 Barcelona, Spain

## Abstract

At calving, 2,213 Holstein cows from 5 farms were enrolled in a study to establish potential associations between postcalving health disorders and reproductive performance and to determine the effectiveness in achieving pregnancy at first artificial insemination (AI) and the consequences on the proportion of cows pregnant by 150 DIM when using 2 different reproductive management programs for first service. Within farm, cows were randomly allocated to 2 reproductive schemes: (1) inseminating based on estrus detection with a backup synchronization program (SP) on open cows with DIM ≥ 80 (HD; 1,141 cows, 34% primiparous [PPC]) or (2) after an SP (1,072 cows, 35% PPC). Cows on HD were inseminated when detected in estrus using an accelerometer if DIM >50, and those not inseminated based on estrus by 80 ± 3 DIM (28.8%) were submitted to an Ovsynch protocol, if a corpus luteum (CL) was present, or to the same Ovsynch protocol but with the addition of a progesterone-releasing intravaginal device if CL was absent. Cows on SP were submitted to a simplified double-Ovsynch protocol (the first GnRH injection was omitted) and serviced between 74 and 80 DIM. On average, cows on HD were first inseminated at 72.4 ± 1.63 DIM, whereas those on SP were first inseminated at 78.3 ± 1.63 DIM. At first AI, PPC had a greater (*P* < 0.01) pregnancy at first AI (P/AI; 55.3%) than multiparous cows (MPC; 38.6%). But PPC submitted to SP had the greatest P/AI during the cold season (63.8%), followed by PPC on SP (57.5%) and on HD (54.6%) during the hot season. During the cold season, P/AI in PPC on HD was lower (45.6%) than that of PPC on SP (63.8%); whereas P/AI in MPC was similar among treatments and seasons, except for MPC in HD during the hot season (32.2%) compared with those on SP (41.9%). By 150 DIM, there were no differences in the proportion of pregnant cows between SP and HD. Cows with retained placenta tended to be inseminated later than those that expelled the placenta within 24 h. Cows that experienced metritis tended to have lower P/AI than cows that stayed healthy after calving, but cows on SP that incurred metritis tended to have better P/AI than cows on HD with metritis. Relying on estrus detection may allow for a first AI at lower DIM, but at 150 DIM, the proportions of pregnant cows was similar between the 2 strategies.

## Highlights

• Relying on estrus detection may allow for a first insemination soon after calving.
• At 150 DIM, strict SP or estrus detection with SP backup results in the same proportions of pregnant cows.
• Postpartum disease, such as lameness and hypocalcemia, decreases conception at first AI.

## Graphical Abstract

**Figure:**
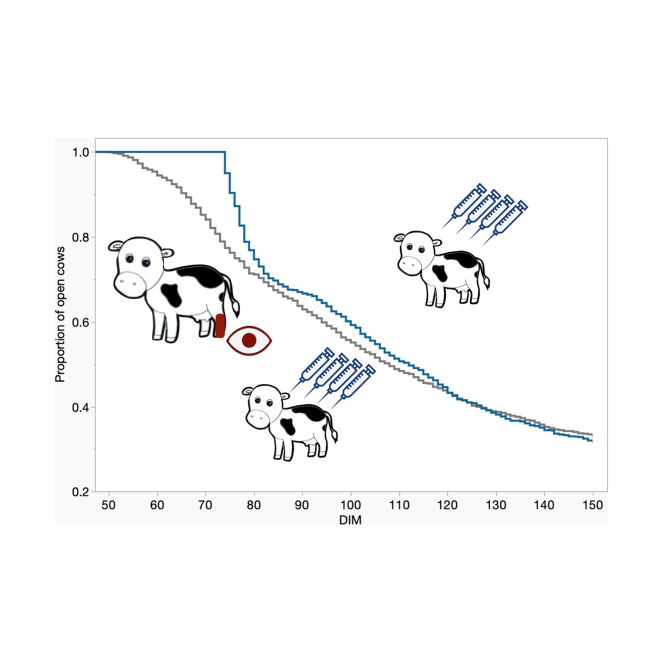
**Summary:** The proportion of lactating cows in a herd and their average milk production are influenced, in part, by the reproductive performance of the animals. Conception can be achieved by detecting natural estrus and subsequently performing artificial insemination (AI) or by synchronizing the estrous cycle and consequently inseminating cows at a fixed time. We studied potential associations between postcalving health disorders and reproductive performance, as well as the effectiveness of using a synchronization program (SP) for fixed-time AI or using natural estrus detection plus an SP as a backup in maximizing the proportion of cows pregnant by 150 days in milk (DIM). Relying on estrus detection led to an initially greater proportion of pregnant cows, but at 150 DIM, there were no differences between the 2 strategies. Cow illustration by Alex Bach; eye icon by Muhammad Rizky Klinsman (Noun Project, https://thenounproject.com/icon/eye-icon-3670607/); syringe icon by Icons Producer (Noun Project, https://thenounproject.com/icon/syringe-2061413/).

Both the proportion of lactating cows in a herd and their average milk production are influenced, in part, by the reproductive performance of the animals. Cows with poor reproductive performance typically have extended lactations or long dry periods, or both, and extended lactations result in lower average daily milk production. Reproductive success is mainly determined by fertility and rate of insemination. Fertility and rate of insemination are affected by postpartum health (Bretzinger et al., 2023), nutrition and genetics (Bach, 2018), and reproductive management (Fricke and Wiltbank, 2022). In terms of reproductive management, conception can be achieved by detecting natural estrus and subsequently performing artificial insemination (**AI**) or by synchronizing the estrus cycle and consequently inseminating cows at a fixed time. Use of fertility programs for first AI increases the number of cows submitted to AI during the first week after the end of the voluntary waiting period and increases the ratio of pregnancies per AI (**P/AI**) by improving circulating progesterone, decreasing exposure to LH pulses, and inducing ovulation of a more optimal-sized follicle with better oocyte quality (Fricke and Wiltbank, 2022). Furthermore, not all cows in estrus are detected by visual observations or by using estrus-detection aids such as accelerometers, and thus the time to reach pregnancy may be delayed because they are not inseminated; however, consumer concerns about using reproductive hormonal treatments in dairy cows are progressively increasing, especially in Europe (Pieper et al., 2016; Lund et al., 2023). Different technology solutions based mainly on accelerometers (i.e., collars, ear or leg tags, rumen boluses) have been developed to assist producers in detecting estrus based on changes in physical activity of the cows. These systems achieve estrus-detection rates >80% with a specificity for detection >90% (Saint-Dizier and Chastant-Maillard, 2012), representing a plausible alternative to reduce hormonal usage in dairy cattle (Gonzalez et al., 2023).

The hypotheses of the current study were that (1) postpartum health disorders may influence reproductive performance of dairy cows and (2) using estrus-detection aids, such as activity monitors, to inseminate cows at first AI may reduce the use of reproductive hormones without affecting reproductive performance. Thus, the objectives of this study were to (1) establish associations between postcalving health disorders and reproductive performance and (2) compare the proportions of pregnancies at first AI and pregnant cows by 150 DIM achieved using a synchronization protocol for fixed-time AI (**TAI**) vs. inseminating based on natural estrus detection and submitting cows to TAI if no estrus was detected.

All experimental procedures were evaluated and approved by the Animal Care Committee of the University of Padova (Padova, Italy) under the protocol number 193758. A 2-sided power analysis conducted with JMP (version 18.1, SAS Institute Inc., Cary, NC) to establish the minimum number of cows needed to detect a difference in conception rate at first insemination between 42% and 48% with a power of 0.80 and an α of 0.05 indicated that a minimum of 1,050 cows per treatment were needed. A total of 2,213 Holstein cows from 5 farms located in the Parmigiano Reggiano area of Italy and milking between 200 and 600 Holstein cows were randomly assigned between July 2021 and December 2022 to 2 reproductive schemes. Herds were selected based on the following parameters: (1) all cows wore automatic estrus-detection devices, (2) the farm's ability to collect data, and (3) the presence of an on-site veterinarian to monitor cows and ensure compliance with reproductive protocols. Three farms used pedometers (AfiTag, SAE Afikim, Kibbutz Afikim, Israel), and 2 herds used collars (Alpro, DeLaval International AB, Tumba, Sweden). Within farm, randomization was conducted using Python version 3.12 and the module random, and treatments were recorded in a management software in each farm (DairyComp 305, Valley Agricultural Software, Visalia, CA). The 2 schemes were as follows: (1) inseminating eligible cows (i.e., DIM > 50 ± 3) based on estrus detection using accelerometers or TAI in those cows not seen in estrus by 80 ± 3 DIM (**HD**; 1,141 cows, 34% primiparous [**PPC**]) or (2) inseminating after a synchronization program (**SP**; 1,072 cows, 35% PPC). At 50 ± 3 DIM all cows were checked for the presence of a corpus luteum (**CL**) ≥ 15 mm using transrectal ultrasound to determine the proportion of anovulatory cows by the end of the voluntary waiting period. Insemination was performed twice daily. Cows on HD were inseminated whenever detected in estrus using an accelerometer, provided DIM was >50 ± 3, and those not inseminated based on estrus by 80 ± 3 DIM were checked using ultrasound for the presence of a CL, and those with a CL were synchronized using an Ovsynch protocol (GnRH at d 0 and 9.5; prostaglandin [**PG**] at d 7 and 8, and TAI approximately 16 h after the last GnRH), and those with no CL were treated with a progesterone-releasing intravaginal device (1.55 g of progesterone, PRID Delta; Ceva Salute Animale, Milano, Italy) at d 0, followed by PG at d 7 and 8, GnRH at d 9.5, and TAI approximately 16 h after the last GnRH). Cows on SP received a modified double-Ovsynch treatment, which consisted of an injection of PG (500 µg of cloprostenol, Cevaprost, Ceva Salute Animale, Milano, Italy) between 54 and 60 DIM followed by GnRH (100 μg of gonadorelin diacetate tetrahydrate, Cystoreline; Ceva Salute Animale, Milano, Italy) 3 d later; then 7 d later, the first GnRH (100 μg of gonadorelin diacetate tetrahydrate) of the Ovsynch followed by 2 doses of PG (500 µg of cloprostenol) 7 and 8 d later, and then 24 h after the last PG, a second GnRH (100 μg of gonadorelin diacetate tetrahydrate) followed by AI 16 h thereafter (between 74 and 80 DIM). Thus, first service was targeted to be performed starting at 74 DIM in SP cows, whereas HD cows were eligible for insemination when detected in estrus after 50 ± 3 DIM. Cows were checked for pregnancy by ultrasound at 32 ± 3 and 68 ± 3 d postinsemination. Cows confirmed pregnant at 32 d but open at 68 d postinsemination were classified as having a pregnancy loss. Cows that showed estrus before pregnancy check were re-inseminated. Farms were visited every 2 wk to randomize cows to treatments and to check records of hormone usage for compliance. Initially, there were 1,139 cows enrolled in the SP program, but 67 cows did not fully comply with the SP and were discarded. Pregnancies per AI at first insemination, DIM at first insemination, DIM at pregnancy, and the proportion of pregnancy losses between 32 and 68 d after first insemination were calculated. After first AI, cows detected in estrus were inseminated again. A 32-d pregnancy check was performed on all animals, and cows found open were submitted to a hormonal protocol. All open cows were checked daily by the on-site veterinarian for health disorders. Hypocalcemia was defined as a fresh cow that exhibited signs of recumbency and even low body temperature and heart rate. Lameness was diagnosed based on the presence of an arched back and an abnormal gait. Retained placenta was defined as the failure to expel the fetal membranes within 24 h after calving. Metritis was defined as the presence of an abnormal, often fetid vaginal discharge along with fever (i.e., rectal temperature ≥39.2°C). Clinical ketosis was defined as cows with blood β-OH-butyrate above 3.0 m*M* and clinical signs consisting of apathy and reduced milk production (all cows with clinical signs were tested for blood BHB concentrations using a portable electronic device (Precision Xtra, Abbott, Abbott Park. IL). Clinical mastitis was diagnosed based on visible abnormalities in the udder or milk (or both), reduced milk yield, or presence of other systemic symptoms (i.e., fever).

Cows were classified as calving in the hot season (June–October, 51% of cows) or the cold season (November–May, 49% of cows). Each dependent variable with a binomial distribution, such as P/AI and pregnancy loss, was analyzed using a mixed-effects logistic regression model with a binomial link accounting for the fixed effects of reproductive scheme (HD or SP), parity (first or older), and season (hot or cold), plus their 2- and 3-way interactions, and farm as a random effect, with mean separation among treatments based on odds ratios and their CI; whereas DIM at first AI was analyzed using the same model but without an underlying binomial distribution. Potential differences between reproductive protocols on the proportion of cows pregnant by 150 DIM were analyzed using logistic regression. Also, for each insemination protocol, time-dependent variables such as hazard for pregnancy were analyzed using time-to-event analyses (survival Kaplan–Meier curves) and Cox proportional hazard regression. Cows that left the herd before 225 DIM without a confirmed pregnancy were right-censored (as they could not have been fully confirmed pregnant at 68 ± 3 d postinsemination). In total there were 57 and 45 cows in HD and SP that left the herds with a median days of lactation of 176 and 181, respectively. There were 16 and 10 cows in HD and SP that did not reach 150 DIM, respectively. The denominator to calculate the percentage of cows pregnant by 150 DIM was the number of cows enrolled in the study. Last, the potential association between incidence of each postpartum disease and reproductive performance was assessed using logistic regression models accounting for the random effect of herd and the fixed effect of health disorder, calving season, reproductive protocol, and their 2- and 3-way interactions.

The proportion of cows with a CL at 50 ± 3 DIM (69.6% ± 0.91%) did not differ (*P* = 0.23) between cows in the 2 reproductive protocols. A proportion (28.8%, n = 329) of cows in the HD protocol were not detected in estrus within the first 80 ± 3 DIM and had to be submitted to a hormone-assisted protocol as described previously. The reason for this proportion of cows that were not found in estrus is probably the result of the combination of the sensitivity of the automatic devices to detect estrus, which ranges between 60% and 90% (Borchardt et al., 2025), and the proportion of cows that are commonly found in anestrus within the first 60 DIM, which is about 20% (Borchardt et al., 2025). At 80 DIM, 72 cows in the HD group (21.9% relative to cows in HD that were not detected in estrus) did not have a CL. Cows in the HD protocol inseminated at estrus were more likely (odds ratio [**OR**] = 1.32; CI = [1.05–1.67]) to conceive than HD cows submitted to TAI, with the P/AI for HD cows that were inseminated in estrus being 40.7% and that for cows submitted to TAI being 36.2%. These figures are difficult to compare because of the confounding factors of lactation stage and inherent characteristics of the cows. Nevertheless, they are comparable to P/AI observations reported between inseminating cows based on estrus detection and hormone intervention or solely using SP (Dolecheck et al., 2016).

Table 1 depicts reproductive performance of cows as affected by reproductive scheme, calving season, and parity. Two- and 3-way interactions are omitted from Table 1 because none were significant for any of the variables except for P/AI at first AI that was affected by an interaction (*P* = 0.03) between parity, insemination protocol, and season (Figure 1B). As expected, P/AI was greater (*P* < 0.01; OR = 1.38, CI = [1.08–2.23] in PPC (55.3%) than in multiparous cows (**MPC**; 38.6%). The parity effect observed in the current study may be, at least, partly due to incomplete luteal regression in MPC along with lower circulating steroid concentrations (Sartori et al., 2004). It has been proposed that PPC have a greater luteolysis rate than MPC and that this contributes to better fertility (Martins et al., 2011). Nevertheless, during the hot season, MPC in the HD protocol had a lower (*P* < 0.05) P/AI at first AI (32.2%) than MPC in SP (41.9%), whereas in the cold season, MPC in HD had a P/AI at first AI (39.0%) similar to MPC in SP (42.0%). The decrease in P/AI in MPC in the HD protocol during the hot season compared with SP could be related to the quality of the follicle. Several studies have described reductions in P/AI under high environmental temperatures due to reduced ovulation rate, follicle growth, oocyte quality, and endometrial function (Wolfenson and Roth, 2019). In contrast, PPC submitted to SP had the greatest P/AI during the cold season (63.8%), followed by PPC on SP (57.5%) and on HD (54.6%) during the hot season, with PPC in HD during the cold season having the lowest (*P* < 0.05) P/AI at first AI (45.6%). Differences in P/AI observed between reproductive protocols could be related, in part, to differences in DIM at first service (Ferguson, 1991). Average DIM at first AI was lower (*P* < 0.01) in HD than in SP cows, regardless of parity and calving season (Table 1). The fact that HD cows were inseminated sooner than SP cows was expected because AI was initiated after 50 ± 3 DIM in HD cows and it did not start until 74 ± 3 DIM in SP cows.

**Table 1:** Reproductive performance of cows submitted to a program based on estrus detection using accelerometers or fixed-time insemination in open cows with >80 DIM (HD) or to a hormonal synchronization program (SP)

Item	Treatment		*P-*value		
	HD	SP	Treatment	Parity	Season
DIM at first AI (± SE)	72.4 ± 1.63	78.3 ± 1.63	<0.01	0.37	0.81
P/AI at first AI,^1^ %^2^	39.4 (n = 1,141)	48.5 (n = 1,072)			
	OR^2^ = 0.81 (0.43–0.97)		<0.01	<0.01	0.36
Pregnancy loss at first AI, %	9.37	8.26			
	OR^2^ = 1.13 (0.59–1.35)		0.76	0.03	0.17
Cows pregnant at ≤150 DIM, %	67.6	71.9			
	OR^2^ = 0.96 (0.71–1.15)		0.13	<0.01	0.19

1 n = the number of cows inseminated in each treatment. There were 329 cows (28.8%) in the HD protocol that were inseminated after receiving a hormonal treatment because they were not detected to be in estrus. The interaction between treatment, parity, and season was significant (*P* < 0.05) for P/AI at first AI, and results are described in the text.

2 OR using SP as a reference (95% CI in parentheses).

**Figure 1:**
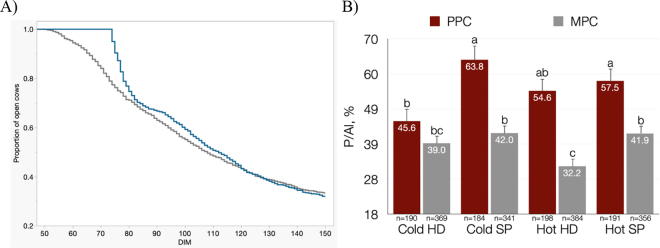
(A) Survival curve for pregnancy for cows on HD (n = 1,141; gray line) or SP (n = 1,072; blue line) up to 150 DIM and (B) interaction between reproductive protocol, parity, and season on pregnancies per insemination at first insemination. HD = first insemination on natural estrus or with an SP if not seen in estrus by 80 ± 3 DIM; SP = first insemination after an SP; PPC = primiparous cows; MPC = multiparous cows; Cold = cold season, June to October; Hot = hot season, November to May. After first insemination, all cows were on a combination of estrus detection and hormonal treatments. The overall hazard for pregnancy during the first 150 DIM did not differ between reproductive schemes. Error bars represent SEM. Bars with different letters (a, b, c) differ at *P* < 0.05.

Pregnancy losses after first AI (i.e., cows that were found pregnant at 32 ± 3 d but were open when rechecked at 68 ± 3 d) did not differ between reproductive protocols or calving season (Table 1), and as expected, losses were greater (*P* < 0.01; OR = 1.73, CI = [1.06–2.80]) in MPC (10.9%) than in PPC (6.6%). Figure 1A shows the proportion of cows open during the first 150 DIM. The median time to pregnancy up to 150 DIM for HD cows was 110 DIM (CI 107–113 DIM) and for SP cows was 114 DIM (CI 111–117 DIM), and the hazard ratios (0.94; CI = 0.86–1.04) for pregnancy (i.e., the relative risk for pregnancy between SP and HD using HD as a reference) did not differ (*P* = 0.15). Thus, despite an initially greater proportion of cows pregnant in the HD protocol, the overall reproductive outcomes of HD and SP did not differ because both time to pregnancy and proportion of pregnant cows by 150 DIM was the same, providing support for using a combination of estrus-detection aids and hormonal treatments to achieve similar reproductive outcomes with lower hormonal usage than implementing reproductive schemes solely based on TAI.

Incidence of health disorders recorded in this study corresponds to the definition used for each disorder described previously, and results might be different had other criteria been used; nevertheless, diagnosis criteria were consistent across farm and treatments. The recorded incidence of retained placenta in this study was 7.5%. Cows that experienced retained placenta tended (*P* = 0.09) to be inseminated later (77.5 ± 1.76 DIM) than those that expelled the placenta within 24 h after calving (75.2 ± 1.60 DIM) regardless of the applied reproductive protocol (*P* = 0.39). Although P/AI at first AI was not affected by the incidence of retained placenta (*P* = 0.76), pregnancy loss after first AI was greater (*P* < 0.01; OR = 3.09, CI = [1.68–5.66]) in cows that incurred retained placenta (23.4% loss) than in those that did not (8% loss), regardless of the reproductive protocol (*P* = 0.29). The incidence of clinical metritis (6%) in the current study was lower than in previous reports (Genís et al., 2018; Molinari et al., 2022). Nevertheless, there was an interaction (*P* < 0.05) between reproductive strategy and incidence of metritis on P/AI at first service. Cows that experienced metritis tended (*P* = 0.09) to have lower P/AI (OR = 0.76, CI = [0.55–1.05]) than cows not diagnosed with metritis after calving, and cows on SP that developed metritis tended (*P* = 0.09) to have better P/AI (OR = 2.9, CI = [1.51–5.56]) than cows on HD that had metritis. Several studies have reported that metritis has a negative effect on reproductive performance (Giuliodori et al., 2013; Vieira-Neto et al., 2014), but to our knowledge this is among the first studies showing a potential advantage of using SP on cows with metritis. Nevertheless, the incidence of metritis in the current study was low, and a larger sample size may be needed to further confirm the tendencies obtained in this study. Clinical ketosis was recorded in only 1.65% of the cows, and there were no associations between incidence of clinical ketosis and P/AI at first AI (*P* = 0.46). Some studies have described negative associations between ketosis and reproductive performance, especially subclinical ketosis (McArt et al., 2012; Raboisson et al., 2014; Rutherford et al., 2016). The incidence of clinical ketosis in the current study was lower than that in previous reports, where incidence typically ranged between 8% and 12% (Rodríguez et al., 2017; Wisnieski et al., 2019), and perhaps the lack of association between ketosis and reproductive performance could be attributed, in part, to the low incidence of disease observed in this study. Overall incidence of mastitis was 17.2%, but incidence between calving and first AI was 9.8%. There was no association (*P* = 0.52) between incidence of mastitis before first AI and subsequent P/AI. Ramos et al. (2022) reported a negative association between mastitis before first AI and fertility, with a similar incidence of mastitis (∼11%) as in the current study. In the current study, 12% of cows were reported to be lame after calving, and those cows had a lower (*P* < 0.01) P/AI at first AI (OR = 0.70; CI = [0.54–0.71]) than cows with good foot health, regardless of the reproductive management. This result is in line with previous studies describing reductions in fertility associated with lameness (Tsousis et al., 2022). Last, 2.5% of cows experienced clinical hypocalcemia, and these animals had a lower (*P* < 0.05) P/AI at first AI (OR = 0.52, CI = [0.29–0.94]) than those that did not develop clinical hypocalcemia after calving regardless of the reproductive protocol (*P* = 0.98). The reduction in P/AI at first AI in cows experiencing milk fever has been reported previously (Venjakob et al., 2018), and it has been linked to increased risk of uterine prolapses (Mulligan et al., 2006) and retained placenta (Gild et al., 2015); this latter observation was also confirmed in this study because clinical hypocalcemia was positively associated with incidence of retained placenta (*P* < 0.01; OR = 27.7, CI = [10.29–33.94]).

In summary, relying on estrus detection may allow for a first insemination at lower DIM, and may yield an initially greater proportion of pregnant cows, but at 150 DIM, synchronization protocols result in overall similar proportions of pregnant cows. Thus, implementing full SP described herein only in the first insemination is not sufficient to increase the proportion of cows pregnant by 150 DIM when compared with a combination of natural estrus detection and synchronization as a backup. In addition, postpartum health disorders such as hypocalcemia and lameness may negatively affect conception rates after calving.
